# Supplementary material for: CryoEM structure of ALK2:BMP6 reveals distinct mechanism that allow ALK2 to interact with both BMP and activin ligands
Source: Proc Natl Acad Sci U S A. 2025 Aug 25;122(35):e2502788122. doi: 10.1073/pnas.2502788122 (PMC12415261; doi:10.1073/pnas.2502788122)
Supplement: Supplementary file 1 — Appendix 01 (PDF) [file pnas.2502788122.sapp.pdf]

PNAS

Supporting Information for

CryoEM structure of ALK2:BMP6 reveals distinct mechanisms that allow ALK2 to interact with both BMP and Activin ligands

Erich J. Goebel[1,2], Senem Aykul[1], Warren W. Hom[1], Kei Saotome[2], Aris N. Economides[1], Matthew C. Franklin[2], Vincent J. Idone[1]

[1]Connective Tissue Diseases Therapeutic Focus Area, Regeneron Pharmaceuticals, Tarrytown, NY 10591
[2]Structural Biology, Regeneron Pharmaceuticals, Tarrytown, NY 10591

Corresponding Author:
Erich J. Goebel
Erich.Goebel@regeneron.com

This PDF file includes:

Figures S1 to S8
Tables S1 to S2

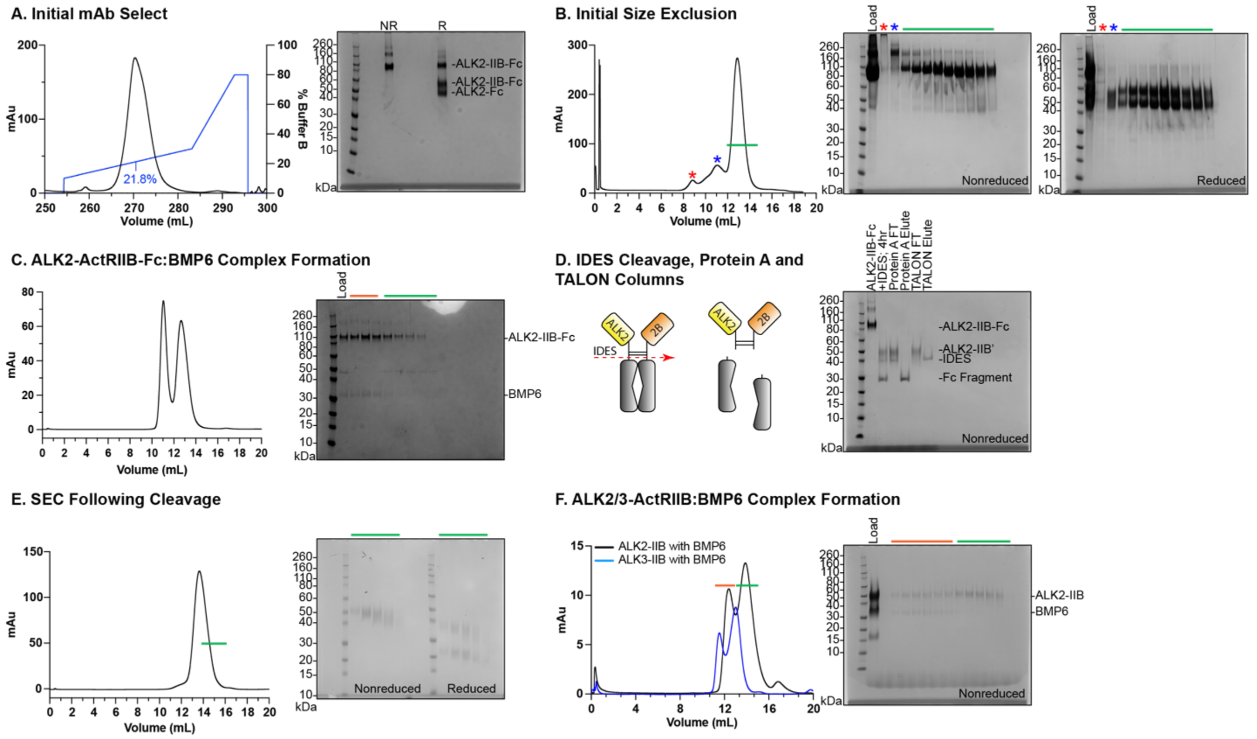

Supplemental Figure 1. Representative biochemistry, purification, and complex formation of ALK2-ActRIIB:BMP6. A. Anti-Fc column (mAb Select) run to purify ALK2-ActRIIB-Fc from conditioned media. SDS PAGE gel displays non-reduced (NR) and reduced (R) samples from major peak. **B.** Size exclusion to purify ALK2-ActRIIB-Fc and corresponding nonreduced and reduced SDS PAGE gels. *Red* and *blue* Asterisks correspond to samples from respective peaks along with samples from throughout the major peak represented by the *green* bar. C. Complex formation and subsequent size exclusion of ALK2-ActRIIB-Fc:BMP6. Complex formation confirmed through SDS PAGE. D**.** IDES cleavage schematic and SDS PAGE gel displaying flowthrough (FT) and elution products following sequential Protein A and TALON gravity columns. **E.** Size exclusion to purify ALK2-ActRIIB to monodispersity. **F.** Complex formation and subsequent size exclusion between ALK2-ActRIIB (*black*). and ALK3-ActRIIB (*blue*) with BMP6. Samples on SDS PAGE gel correspond to ALK2-ActRIIB trace (*black*) and are from the corresponding peak with colored band above.

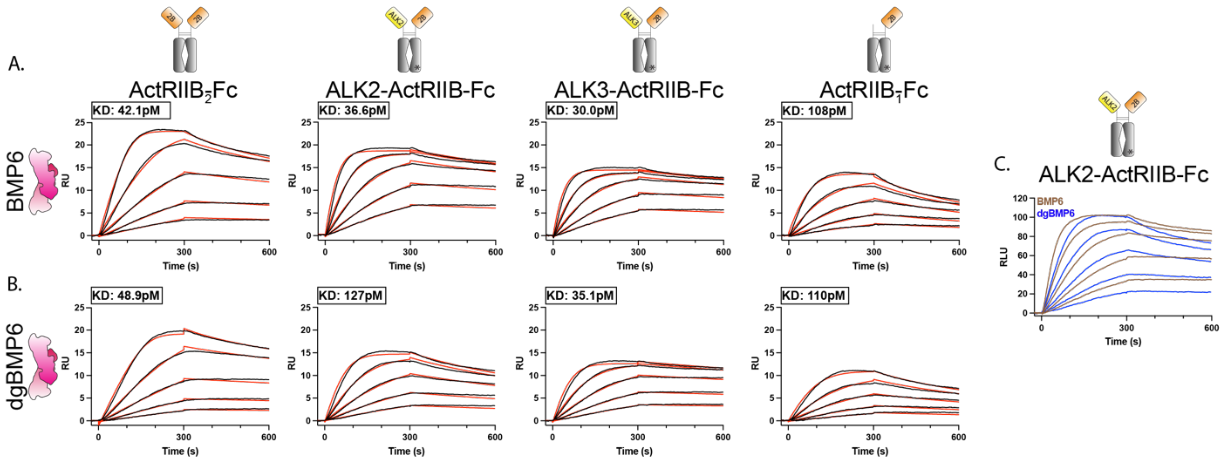

Supplemental Figure 2. Heterodimeric traps, ALK2-ActRIIB-Fc and ALK3-ActRIIB-Fc bind BMP6 with high affinity. A. SPR sensorgrams of BMP6 and B. deglycosylated BMP6 (dgBMP6) binding to anti-human antibody-captured ActRIIB$_2$-Fc, ALK2-ActRIIB-Fc, ALK3-ActRIIB-Fc, or ActRIIB$_1$-Fc. Sensorgrams (*black lines*) are overlaid with fits to a 1:1 interaction model with mass transport limitations (*red lines*). C. Overlay of the binding data for both ligands, following normalization for direct comparison, against ALK2-ActRIIB-Fc. Each experiment was performed in triplicate and the kinetic parameters are summarized in Table S1.

Analyte	Ligand	k_a ($M^{-1}s^{-1}$)	k_d (s^{-1})	K_D (pM)[a]
BMP6	$ActRIIB_2$-Fc	$3.17 \pm 1.23 \times 10^7$	$1.19 \pm 0.37 \times 10^{-3}$	42.1 ± 19.0
BMP6	ALK2-ActRIIB-Fc	$1.43 \pm 0.24 \times 10^7$	$5.28 \pm 1.27 \times 10^{-4}$	36.6 ± 3.10
BMP6	ALK3-ActRIIB-Fc	$1.39 \pm 0.21 \times 10^7$	$4.23 \pm 1.20 \times 10^{-4}$	30.0 ± 4.70
BMP6	$ActRIIB_1$-Fc	$2.16 \pm 0.05 \times 10^7$	$2.34 \pm 0.61 \times 10^{-3}$	108 ± 26.3
**Analyte**	**Ligand**	**k_a ($M^{-1}s^{-1}$)**	**k_d (s^{-1})**	**K_D (pM)[a]**
dgBMP6	$ActRIIB_2$-Fc	$3.12 \pm 0.34 \times 10^7$	$1.52 \pm 0.01 \times 10^{-3}$	48.9 ± 6.00
dgBMP6	ALK2-ActRIIB-Fc	$1.16 \pm 0.07 \times 10^7$	$1.47 \pm 0.10 \times 10^{-3}$	127 ± 1.88
dgBMP6	ALK3-ActRIIB-Fc	$1.23 \pm 0.04 \times 10^7$	$4.32 \pm 0.11 \times 10^{-4}$	35.1 ± 0.47
dgBMP6	$ActRIIB_1$-Fc	$1.70 \pm 0.26 \times 10^7$	$1.86 \pm 0.40 \times 10^{-3}$	110 ± 14.8

All kinetic parameters were analyzed using the Biacore T200 evaluation software using a 1:1 binding model.

Supplemental Table 1. SPR Kinetic Analysis.

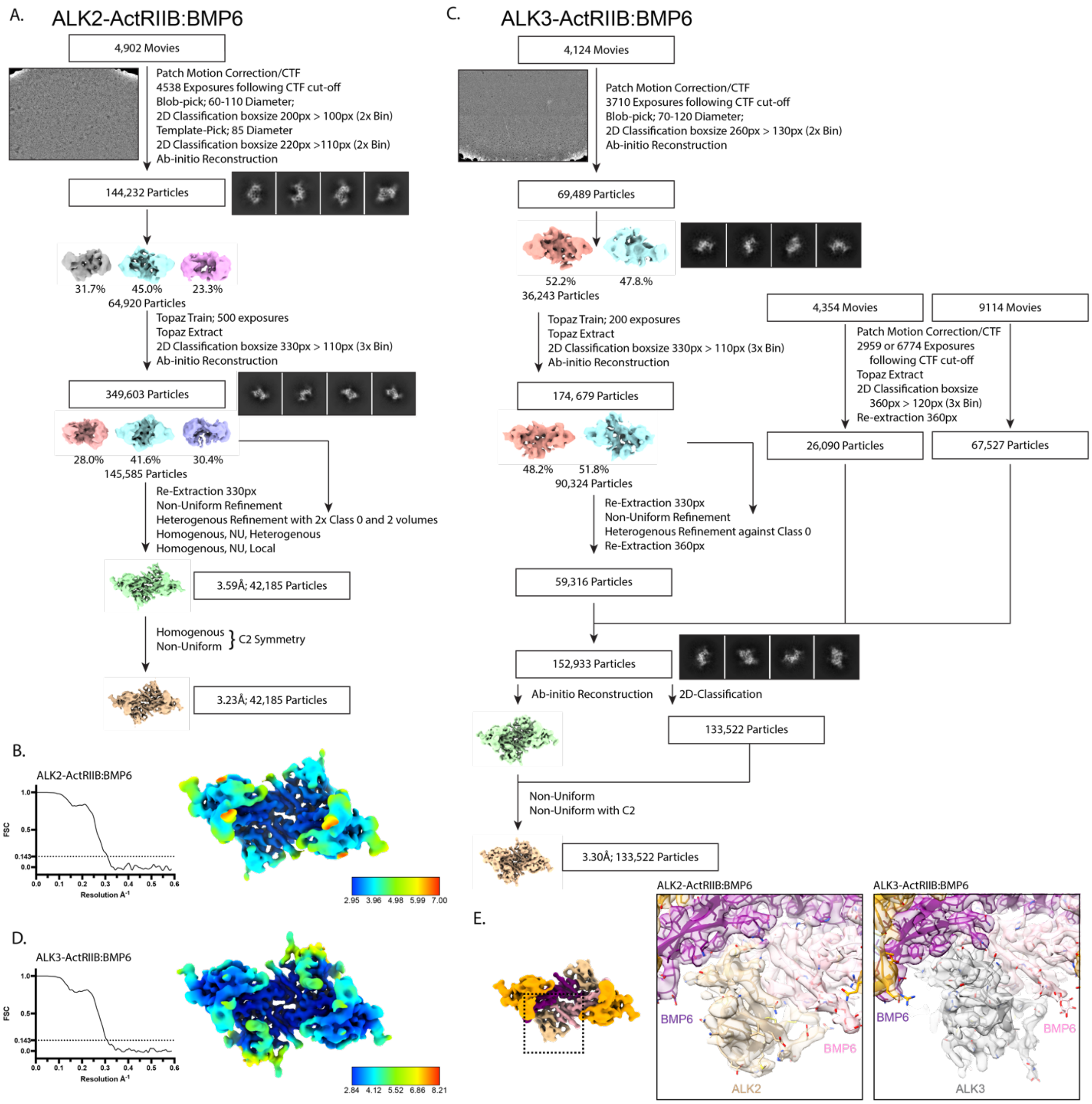

Supplemental Figure 3. CryoEM data processing and reconstruction of BMP6 in complex with ALK2-ActRIIB and ALK3-ActRIIB. A. Data processing flow chart for ALK2-ActRIIB:BMP6. **B.** FSC curve output and local resolution map by cryoSPARC for ALK2-ActRIIB:BMP6. **C.** Data processing flow chart for ALK3-ActRIIB:BMP6. **D.** FSC curve output and local resolution map by cryoSPARC for ALK3-ActRIIB:BMP6. **E.** Overlay of sharpened maps and model focused on the respective type I receptors.

	ALK2-ActRIIB:BMP6 (9N4K)	ALK3-ActRIIB:BMP6 (9MIR)
**Data Collection and Processing**		
Magnification	105,000	105,000
Voltage (kV)	300	300
Electron exposure ($e^{-}/Å^{2}$)	~40	~40
Defocus Range (µm)	-0.8 to -2.4	-1 to -3.4
Pixel size (Å)	0.839	0.839
Number of Movies	4902	13,443 (3710 + 2959 + 6774)
Initial number of particles	1,820,762	3,218,335 (778,719 + 752,555 + 1,686,061)
Particles selected after 2D classification	349,603	152,933 (59,316 + 26,090 + 67,527)
Final selected particles	42,185	133,522
Symmetry imposed	C2	C2
Map resolution (Å)	3.2	3.3
FSC threshold	0.143	0.143
**Refinement**		
Initial Model used	PDBs: 6MAC, 6OMO, 7YRU	PDBs: 2GOO, 6MAC, 6OMO
**Model Composition**		
Non-hydrogen atoms	4578	4822
Protein Residues	552	576
Ligands	BMA: 4; NAG: 10	BMA: 4, NAG: 14
**R.m.s Deviations**		
Bond lengths (Å)	0.003	0.003
Bond Angles (°)	0.759	0.726
**Validation**		
MolProbity Score	1.67	1.73
Rotamer Outliers (%)	0.00	0.39
Clash Score	4.44	5.39
**Ramachandran Plot**		
Favored (%)	92.96	93.26
Allowed (%)	7.04	6.74
Disallowed (%)	0.00	0.00
**Deposition ID**		
PDB	9N4K	9MIR
EMDB	48883	48301

Supplemental Table 2. CryoEM data, structure refinement and validation.

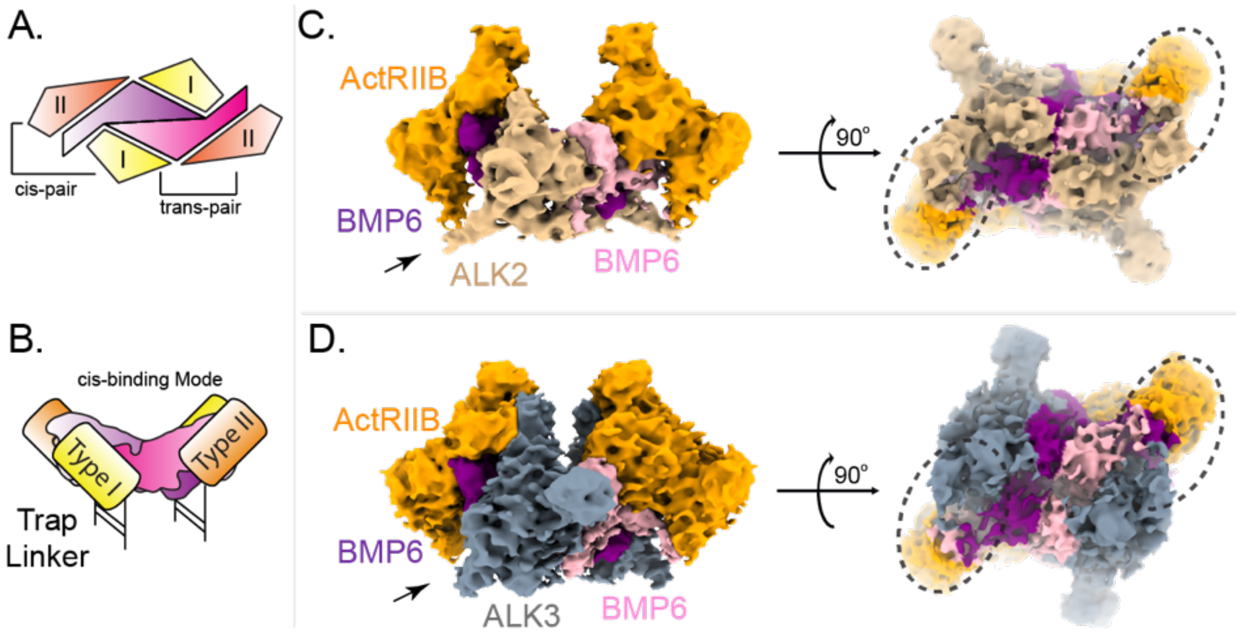

Supplemental Figure 4. ALK2-ActRIIB and ALK3-ActRIIB bind BMP6 in a cis-manner. A. Schematic displaying the two potential pairs of receptors in the heterotetrametric complex. **B.** Schematic displaying the Fc-cleaved Trap binding ligand in a cis-binding mode. **C. and D.** CryoEM maps displaying weakly resolved signal (indicated by black arrows) for intact antibody-hinge tether between the cis-pair of receptors in ALK2-ActRIIB:BMP6 (**C.**) and ALK3-ActRIIB:BMP (**D.**).

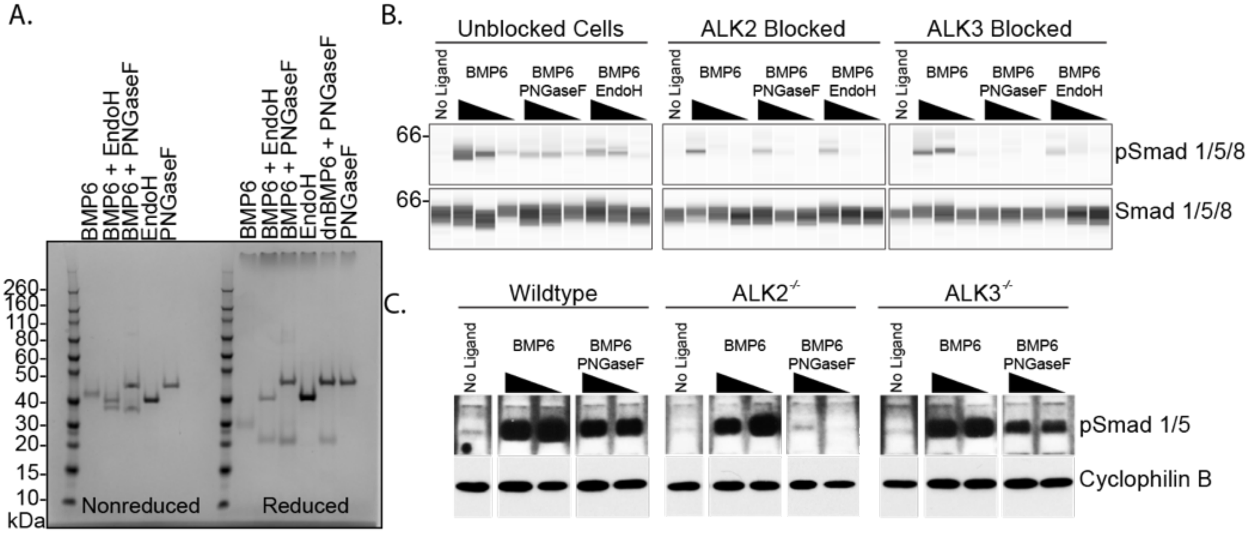

Supplemental Figure 5. Deglycosylation of BMP6 results in a loss of ALK2, but not ALK3 signaling. A. SDS PAGE displaying differential deglycosylation of BMP6 following treatment with either EndoH or PNGaseF in both nonreducing (*Left*) and reducing *(Right*) conditions. Denatured BMP6 (dnBMP6) shown to demonstrate complete deglycosylation. B. SimpleWestern probing whole cell lysates from HEK cells following treatment (30 nM, 10 nM and 3 nM) with BMP6, PNGaseF treated BMP6 or EndoH treated BMP6 with supplementation of ALK2 blocking antibody (*middle;* Regn5168) or ALK3 blocking Fab (*right;* AbD01564*)*. C. Western probing lysates from wildtype ($ALK2^{[R206H]FlEX/+}$), $ALK2^{-/-}$, and $ALK3^{-/-}$ ($ALK3^{-/-}$; $ALK2^{[R258H]FlEx/+}$) mES cells following treatment (100 nM, and 10 nM) with either BMP6 or PNGaseF treated BMP6.

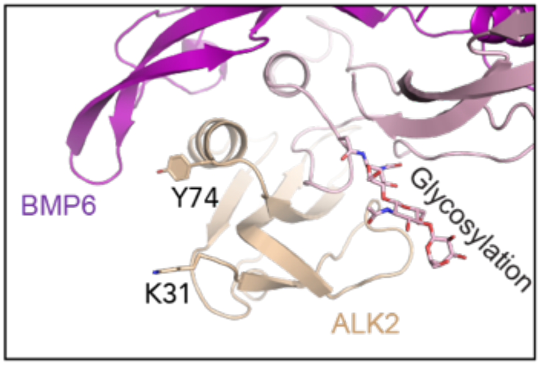

Supplemental Figure 6. Structural zoom into ALK2-ActRIIB:BMP6 that displays the positioning of residues Tyr^{74} and Lys^{31}, previously modeled to interact with the glycosylation on BMP6 (1).

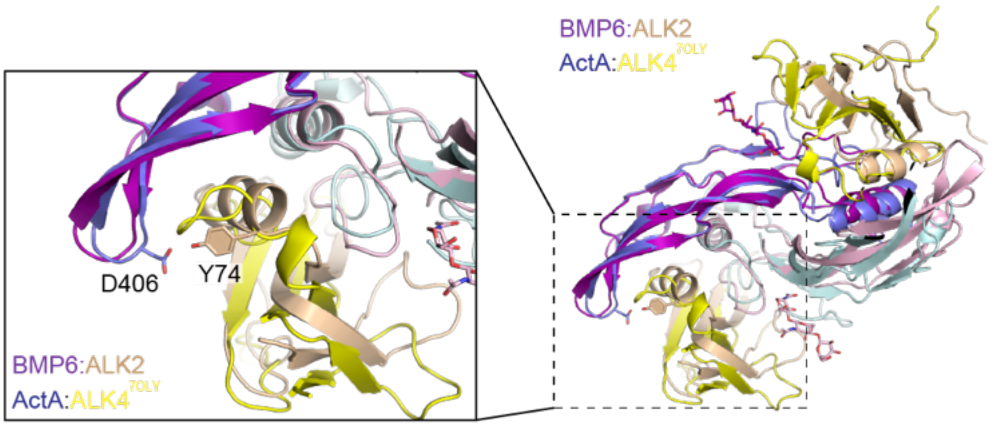

Supplemental Figure 7. Alignment of structures: ALK2-ActRIIB:BMP6 and ALK4-ActRIIB:ActA (PDB code: 7OLY (2)). ActRIIB is hidden in this representation and alignment performed on darker colored ligand monomers. Zoom in highlights positioning of the ALK2 β4β5 loop and residue, Y74.

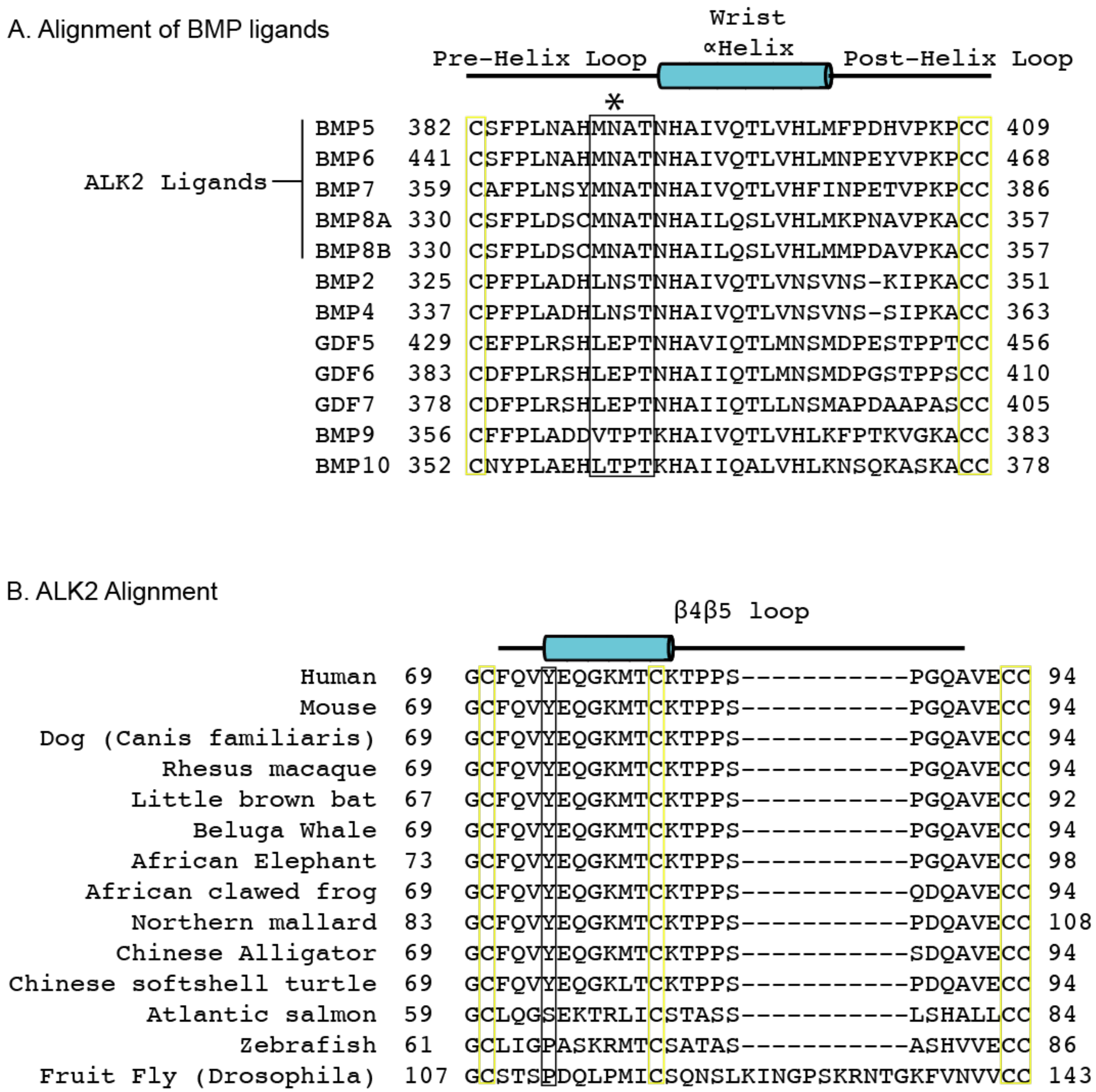

Supplemental Figure 8. Sequence alignment of BMP wrist-helix regions and ALK2 across Species. A. Alignment of human BMP ligands, highlighting the wrist helix region. Asterisk denotes N-linked glycosylation site, while flanking residues are boxed in *black.* Conserved cystine positions flanking the wrist helix region are boxed in *yellow*. B. Alignment of the β4β5 loop region from the sequences of several species. ActA-critical and well-conserved residue, Tyr^{74} boxed in *black*.

SI References.

1. S. Saremba, *et al.*, Type I receptor binding of bone morphogenetic protein 6 is dependent on N-glycosylation of the ligand. *FEBS J.* **275**, 172–183 (2008).

2. E. J. Goebel, *et al.*, Structures of activin ligand traps using natural sets of type I and type II TGFβ receptors. *iScience* **25**, 103590 (2022).
